# What impact does nursing care left undone have on patient outcomes? Review of the literature

**DOI:** 10.1111/jocn.14058

**Published:** 2017-10-16

**Authors:** Alejandra Recio‐Saucedo, Chiara Dall'Ora, Antonello Maruotti, Jane Ball, Jim Briggs, Paul Meredith, Oliver C Redfern, Caroline Kovacs, David Prytherch, Gary B Smith, Peter Griffiths

**Affiliations:** ^1^ National Institute for Health Research (NIHR) Collaboration for Applied Health Research and Care (CLAHRC) Wessex University of Southampton Southampton UK; ^2^ Faculty of Health Sciences University of Southampton Southampton UK; ^3^ Dipartimento di Scienze Economiche Politiche e delle Lingue Moderne – Libera Università Maria Ss Assunta Roma Italy; ^4^ School of Computing University of Portsmouth Portsmouth Hampshire UK; ^5^ TEAMS Centre Portsmouth Hospitals NHS Trust Portsmouth UK; ^6^ Faculty of Health and Social Sciences University of Bournemouth Bournemouth Dorset UK

**Keywords:** care left undone, missed care, nurse staff, patient outcomes, safe staffing levels, unfinished care

## Abstract

**Aims and objectives:**

Systematic review of the impact of missed nursing care on outcomes in adults, on acute hospital wards and in nursing homes.

**Background:**

A considerable body of evidence supports the hypothesis that lower levels of registered nurses on duty increase the likelihood of patients dying on hospital wards, and the risk of many aspects of care being either delayed or left undone (missed). However, the direct consequence of missed care remains unclear.

**Design:**

Systematic review.

**Methods:**

We searched Medline (via Ovid), CINAHL (EBSCOhost) and Scopus for studies examining the association of missed nursing care and at least one patient outcome. Studies regarding registered nurses, healthcare assistants/support workers/nurses’ aides were retained. Only adult settings were included. Because of the nature of the review, qualitative studies, editorials, letters and commentaries were excluded. PRISMA guidelines were followed in reporting the review.

**Results:**

Fourteen studies reported associations between missed care and patient outcomes. Some studies were secondary analyses of a large parent study. Most of the studies used nurse or patient reports to capture outcomes, with some using administrative data. Four studies found significantly decreased patient satisfaction associated with missed care. Seven studies reported associations with one or more patient outcomes including medication errors, urinary tract infections, patient falls, pressure ulcers, critical incidents, quality of care and patient readmissions. Three studies investigated whether there was a link between missed care and mortality and from these results no clear associations emerged.

**Conclusions:**

The review shows the modest evidence base of studies exploring missed care and patient outcomes generated mostly from nurse and patient self‐reported data. To support the assertion that nurse staffing levels and skill mix are associated with adverse outcomes as a result of missed care, more research that uses objective staffing and outcome measures is required.

**Relevance to clinical practice:**

Although nurses may exercise judgements in rationing care in the face of pressure, there are nonetheless adverse consequences for patients (ranging from poor experience of care to increased risk of infection, readmissions and complications due to critical incidents from undetected physiological deterioration). Hospitals should pay attention to nurses’ reports of missed care and consider routine monitoring as a quality and safety indicator.

**What does this article contribute to the wider global clinical community?**

- Nursing staff and patients indicate instances where care delivered or received is incomplete and suboptimal when staffing levels are inadequate.
- The negative impact on patient outcomes resulting from missed care highlights the significance of exploring further the factors that affect the completion of nursing activities.

## 1 BACKGROUND

The association between inadequate quality of nursing care and patient harm has been highlighted as an issue in numerous reports into failings in National Health Service (NHS) hospitals in England (Keogh, 2013). Indeed, failure to ensure adequate nurse staffing levels has frequently been cited as a contributing factor (Luettel, Beaumont, & Healey, 2007; Smith, 2010). Delayed or unfinished care, more broadly identified as *missed care*, encompasses all aspects of clinical, emotional or administrative nursing care that have only been partially completed, were delayed or were not completed at all. The terminology used to refer to missed care varies slightly with the instruments used in the studies of the field. In some instances, missed care is viewed as a form of *care rationing* (Jones, Hamilton, & Murry, 2015), or care left undone (Ausserhofer et al., 2014), while in others, the focus is on *unmet patient need* (Lucero, Lake, & Aiken, 2009). Most evidence of missed care comes from self‐reported nursing or patient questionnaires (Jones et al., 2015).

The current literature on missed care provides mounting evidence of the pervasive nature of the problem and, more importantly, the threat it poses to patient safety. Patient outcomes reported in the missed care literature, which have been associated with quality of care delivered, include hospital‐acquired infections, discharge planning, mortality, falls, patient mobilisation, feeding, psychological and emotional support (Cho, Kim, Yeon, You, & Lee, 2015; Kalisch, 2006; Kalisch, Tschannen, & Lee, 2011, 2012; Papastavrou, Andreou, & Efstathiou, 2014; Schubert, Clarke, Aiken, & de Geest, 2012). Likely factors that influence care prioritisation and completion include the time that is required to complete a care task and the immediate effect that delaying or missing this task might have on patients (Kalisch, 2006).

Studies exploring missed care under the *implicit rationing* approach have found that nursing activities related to surveillance are among the top five most frequently left undone (Jones et al., 2015; Rochefort & Clarke, 2010; Schubert et al., 2012). These findings resonate with analysis by Smith (2010) about the acute problem regarding frequency of physiological observations. Smith proposes that the problem might lie in the levels of trained staff, suggesting that more nursing staff on duty might provide better surveillance, resulting in reduced deterioration, cardiac arrest and failure‐to‐rescue.

Resource adequacy and nurse staffing have been reported as key environment factors influencing the incidence and prevalence of missed care. A considerable body of evidence supports the hypothesis that lower levels of registered nurses on duty increase the likelihood of patients dying on hospital wards (Griffiths et al., 2016; Needleman et al., 2011) and the risk of many aspects of care being either delayed or left undone (Ausserhofer et al., 2014). Guidelines on safe staffing published by the National Institute for Care and Health Excellence (NICE) highlighted the need for more evidence and indicators to determine safe nurse staffing levels, and studies to determine the extent to which they are achieved in practice. Furthermore, NICE proposed that missed care could be used as a “red flag” to warn of inadequate staffing levels and, as a result, be a potential useful indicator of the quality of nursing services (National Institute for Health and Care Excellence (NICE), 2014).

In this systematic review, we searched for quantitative studies reporting associations between missed care and patient outcomes in acute hospital and nursing homes, where care is delivered by nursing staff. We then assessed the evidence of the short‐ and long‐term effects that missed care has on patients.

## 2 AIM

To conduct a systematic review of the impact of missed nursing care on outcomes in adults on acute hospital wards and in nursing homes.

## 3 METHOD

Medline (via Ovid), CINAHL (EBSCOhost) and Scopus were searched for studies examining the association of missed nursing care and at least one patient outcome. We included primary research where missed care was not treated as the outcome measure. Studies regarding care delivered by registered nurses, healthcare assistants/support workers/nurses’ aides were retained. We included studies conducted in acute hospitals or nursing homes; only adult settings were considered. Only studies with quantitative evidence were retained. Consequently, qualitative studies, editorials, letters and commentaries were excluded. Papers were not excluded on the basis of replicability or generalisability of findings. This review is reported according to the Preferred Reporting Items for Systematic Reviews and Meta‐analyses (PRISMA) guidelines (Moher, Liberati, Tetzlaff, & Altman, 2009).

### 3.1 Search strategy

The search strategy was built using free‐text keywords and medical subject headings, and related to missed nursing care and patient outcomes. Because of the different conceptualisations of missed care in the literature (Jones et al., 2015), we included the following terms: “missed nursing care,” “care rationing,” “care left undone” and “unfinished care.”

Search terms for patient outcomes were as follows: pressure ulcers; falls; catheter‐related and urinary tract infections; venous thromboembolism; patient and/or carer experience (including satisfaction ratings and/or complaints concerning care received); mortality; hospital‐acquired infections; hospital readmissions; medication system errors (i.e., drug administration delayed or missed); quality of health care; and patient safety.

### 3.2 Search results

The search produced 2,430 records. An initial screen of titles was carried out to exclude irrelevant papers, resulting in the retention of 155 titles abstract screened. Following abstract screening, 44 studies were retained for full review, during which 30 studies were excluded due to the following:

- absence of reports of associations between missed care and patient outcomes; *n* = 2
- reports of associations of missed care and staff outcomes instead of patient outcomes; *n* = 2
- unclear definition and assessment of missed care; *n* = 1
- duplication of study as reported in two sources (i.e., doctoral thesis and journal article). The content of the study in a more extended version (i.e., doctoral thesis) was retained; *n* = 1
- Missed care from other health professionals (i.e., not nursing staff); *n* = 1
- Medication errors studied as a missed care process and not as outcomes; *n* = 23

**Figure 1:**
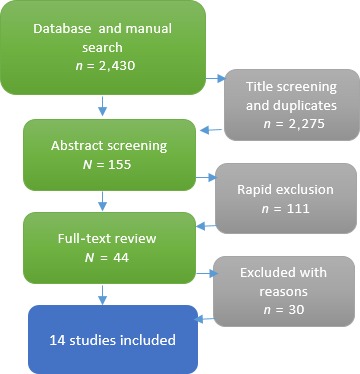
Flow chart of search and inclusion [Colour figure can be viewed at http://wileyonlinelibrary.com]

A total of 14 papers were analysed fully (Figure 1).

### 3.3 Quality appraisal

To assess the quality of the studies, we adapted the National Institute for Health and Care Excellence (NICE) quality appraisal checklist for quantitative studies (National Institute for Health and Care Excellence (NICE), 2014). The quality assessment was expressed in terms of internal and external validity. Internal validity included information on reliability and completeness of the measurements, and ability of the study to control for potential confounding factors. External validity was assessed by appropriate sample size and statistical power. The complete appraisal checklist is available in Appendix.

Quality assessments were performed separately by two reviewers (AR‐S and CDO), and disagreements were resolved by discussion. Most studies were rated as having significant limitations in internal and/or external validity. One study was weak in both aspects of validity, and no study was rated as strong in both. Quality ratings for each study can be found in Table 1.

**Table 1:** Setting, participants and quality appraisal of the included studies

Study	Setting (hospital or units)	Participants (RN = registered nurses) (HCAs = healthcare assistants)	Validity^a^	
			Internal	External
Ausserhofer et al. (2013)	35	1,630 RNs, 997 patients in medical, surgical and mixed medical–surgical units	+	+
Ambrosi et al. (2016)	12	205 RNs, 109 HCAs; 1,464 medical patients	+	−
Ball et al. (2014)	46	2,917 RNs in surgical, medical, surgical/medical units	+	++
Bruyneel et al. (2015)	127	10,733 RNs, 11,549 patients in general surgical and internal medicine units	+	++
Carthon et al. (2015)	419	20,605 RNs 160,930 patients aged 65–90 years old	+	++
Lucero et al. (2010)	168	10,184 RNs, 232,342 general, vascular and orthopaedic surgical patients	−	+
Nelson and Flynn (2015)	63 nursing homes	340 RNs	−	+
Papastavrou, Andreou, Tsangari, et al., (2014)	5	318 RNs, 352 patients in medical and surgical units	−	−
Schubert et al. (2012)	8 (study sample) 71 (comparator group)	1,338 RNs working in a medical, surgical or gynaecological unit, 165,863 patient discharges (study sample), 760 608 patient discharges (comparator group)	++	+
Schubert et al. (2009)	8	1,338 RNs, 779 patients in medical, surgical or gynaecological units	+	+
Schubert et al. (2008)	8	1,338 RNs, 779 patients in medical, surgical or gynaecological units	+	+
Sochalski (2004)	Not specific. Data from state‐wide survey of licensed nurses working in adult care general hospitals were the focus of the study	8,670 RNs working in medical–surgical, intensive care, paediatrics, neonatal intensive care, rehabilitation psychiatry, labour and delivery, operating room, and subacute care	+	‐
Thompson (2014)	550 (2011) 741 (2012)	39,292 RNs (2011); 38,977 RNs (2012) in adult medical, surgical, medical‐surgical units	+	+
Zúñiga et al. (2015)	155 nursing homes	4,311 care workers (registered nurses, licensed practical nurses, nurse aides)	+	+

a Validity scores:

Strong (++): All/most checklist items fulfilled, limitations very unlikely to alter conclusions.

Moderate (+): Some checklist criteria fulfilled, limitations unlikely to alter conclusion.

Weak (−): Few criteria fulfilled, limitations likely to alter conclusions.

## 4 RESULTS

The 14 studies reported a range of outcomes of interest: medication errors; bloodstream infections; pneumonia; urinary tract infections (UTIs); nosocomial infections; patient falls; pressure ulcers; patient and/or carer experience and satisfaction ratings; patient safety; quality of nurse delivered care; critical incidents; adverse events; mortality and 30‐day hospital readmissions.

Most studies measured missed care with nurse or patient surveys that have been widely used in the missed care literature, namely survey from the International Hospital Outcomes Consortium [IHOC]/RN4CAST (Sermeus et al., 2011); MISSCARE (Kalisch, 2006) and the Basel Extent of Rationing of Nursing Care: BERNCA (Schubert, Glass, Clarke, Schaffert‐Witvliet, & De Geest, 2007). Three studies were secondary analyses of the large RN4CAST study conducted across 15 European countries (Ausserhofer et al., 2013; Ball, Murrells, Rafferty, Morrow, & Griffiths, 2014; Bruyneel et al., 2015), where authors analysed and reported data from individual countries. The majority of the studies used nurse or patient reports to capture outcomes, with some studies using administrative data (Table 2).

**Table 2:** Measures of missed care and source of patient outcomes in included studies

Study	Missed care measure	Patient outcome measure & analytical method
Ambrosi et al. (2016)	MISSCARE Survey	In‐hospital mortality. Analysis adjusted for several patient‐level variables (e.g., age, comorbidities, type of admission, pressure ulcer risk score, physical restraints, care received from family members (refer to original publication for full list)
Ausserhofer et al. (2013)	BERNCA‐R Survey	Nurse‐reported medication administration errors; pressure ulcers; patient falls (with injury); urinary tract infections; bloodstream infection (catheter‐related); pneumonia. Analysis was adjusted for patient socio‐demographic characteristics (self‐reported health status and educational level); hospital type (hospital university; centre care hospital; primary care hospital); unit type
Ball et al. (2014)	RN4CAST Survey	Nurse‐reported patient safety and grading quality of nursing care. Analyses were adjusted for intensity originating from variation in patient need
Bruyneel et al. (2015)	RN4CAST Survey	Patients’ overall ratings of the hospital and their willingness to recommend the hospital to friends and family. Analyses were adjusted for hospital characteristics (i.e., size (number of beds), teaching status and technology level [open heart surgery, organ transplantation or both])
Carthon et al. (2015)	Multi‐State Nursing Care and Patient Safety Survey	All‐cause readmission within 30 days of discharge for patients with heart failure. Analyses were adjusted for patient characteristics (age, gender, race, ethnicity, socio‐economic status [SES], length of stay [LOS], discharge disposition and the presence of 27 individual comorbidities); structural hospital characteristics (nurse staffing, teaching status, size, technology capability, ownership, population density, volume of patients with heart failure, Medicare cost‐to‐charge ratio and state); nurse work environment
Lucero et al. (2010)	State‐wide survey of hospital staff nurses in Pennsylvania (no specific name)	Nurse reports of patient received wrong medication or dose; nosocomial infections; falls with injury. Analyses adjusted for patient factors (i.e., illness severity, race and insurance status) and the care environment (i.e., nurse staffing, nursing education, nursing unit type, patient care environment; and hospital bed size, teaching and technology status)
Nelson and Flynn (2015)	Multi‐State Nursing Care and Patient Safety Survey—data from New Jersey only.	Urinary tract infections (UTIs). Analyses adjusted for per cent of residents in nursing home with an indwelling catheter
Papastavrou, Andreou, Tsangari, et al., (2014)	BERNCA Survey	Patient satisfaction. Analyses adjusted for patient and nurse characteristics: age of nurse and patient, patient gender, nurse education, nurse experience (total and in unit) and patient days of hospitalisation
Schubert et al. (2012)	BERNCA Survey	Inpatient mortality rates (constructed from patient discharge method). Risk adjustment, as reported, was adapted from on authors’ earlier work, included adjusting for severity of illness, incorporating data on patient demographic factors (age, sex), procedures (surgery types) and diagnoses, interactions between procedures and diagnoses, and a number of other interaction terms
Schubert et al. (2009)	BERNCA Survey	Nurse‐reported estimates of nosocomial infections; pressure ulcers; medication errors; patient falls; critical incidents; patient satisfaction. No adjustment reported
Schubert et al. (2008)	BERNCA Survey	Nurse‐reported estimates of nosocomial infections; pressure ulcers; medication errors; patient falls; critical incidents; patient satisfaction. Adjusted for nurse education, nurse experience, hospital size, patient health, quality of care, patient self‐care ability, job satisfaction
Sochalski (2004)	State‐wide survey of hospital staff nurses in Pennsylvania (no specific name)	Nurse‐reported quality of care and patient safety. No evidence of adjustment
Thompson (2014)	National Database of Nurse Quality Indicators^®^ (NDNQI^®^ RN) Survey	Pressure ulcers prevalence rate. Adjusted for organisation characteristics (i.e., teaching status, size, location, and Magnet^®^ status), staffing (i.e., RNHPPD), skill mix (i.e., RN hours per patient day/total hours per patient day), and nurse characteristics (i.e., per cent of nurses with a bachelor's degree, per cent certified, average RN tenure)
Zúñiga et al. (2015)	BERNCA‐NH Survey	Care worker reported quality of care. Adjusted for organisation characteristics: language region (German, French, or Italian), profit status (public, private subsidised, private), size (small = 20–49 beds, medium = 50–99 beds, large = 100 and more beds); Unit characteristics: number of beds, percentage of residents with diagnosed dementia or symptoms of dementia; Resident characteristics: mean age per unit, mean length of stay per unit, mean care load; Care worker characteristics: gender, age, educational background

### 4.1 Patient satisfaction

Four studies in hospital settings found missed care significantly decreased patient satisfaction. These findings are summarised in Table 3.

**Table 3:** Studies of missed nursing care, patient satisfaction and quality of care

Study	Context	Associations of missed care and outcomes
Ausserhofer et al. (2013)	Switzerland 132 units (surgical; medical; mixed surgical–medical units)	Rationing of nursing care was associated with patient satisfaction (OR = 0.27; 95% CI = 0.11–0.67)
Bruyneel et al. (2015)	8 European countries (Belgium; Finland; Germany; Greece; Ireland; Poland; Spain; Switzerland) Surgical; medical; mixed surgical–medical units	Clinical care left undone is associated with patients recommending the hospital and patient rating the hospital The amount of care left undone partially mediates the effects of patient‐to‐nurse ratios and work environment on patient recommending the hospital Clinical care left undone mediates the effect of nurse staffing levels on both patient outcomes differently, depending on the proportion of nurses trained to a bachelor's degree
Papastavrou, Andreou, Tsangari, et al., (2014)	Cyprus 10 medical/surgical units	Implicit rationing care was associated with all five dimensions of patient satisfaction: direct nursing care (*p* < .001); technical care (*p* < .001); information (*p* < .001); interpersonal (*p* < .001); indirect nursing care (*p* < .01)
Schubert et al. (2008)	Switzerland 118 units (medical; surgical; gynaecology)	A 0.5‐unit increase in rationing scores was associated with a 37% decrease in the odds of patients reporting satisfaction with the care they received (*p* = .08)—adjusted model
Ball et al. (2014)	England 401 units (medical or surgical)	Correlation between the number of items of missed care and nurses perception of quality of care (polyserial correlation = −0.037, *p* < .001) and nurse overall grading of patient safety on their unit/ward (polyserial correlation = −0.40, *p* < .001)
Sochalski (2004)	USA Number of hospitals not documented 8,670 staff nurses in acute hospitals	There was an association between a poor rating of quality of care and the number of tasks left undone (β = −0.20; *p* < .001)
Zúñiga et al. (2015)	Switzerland 402 units in 155 nursing homes 4,311 care workers (RNs, LPN nursing aides)	Better quality of care was associated with less implicit rationing of caring, rehabilitation, and monitoring (OR 0.34; 95% CI 0.24–0.49); and less rationing of social care (OR 0.80; 95% CI 0.69–0.92)

Bruyneel et al. analysed survey data from 217 hospitals across eight European countries enrolled in the RN4CAST study. Using factor analysis, the authors classified care left undone into two domains—clinical nursing activities and planning/communication activities—and examined the relationships with patient satisfaction. The authors reported a significant association between clinical care left undone (omission of at least one of: adequate patient surveillance, skincare, oral hygiene, pain management, treatments and procedures, timely medication administration, frequently changing the patient's position) and patients recommending the hospital to family and friends (Bruyneel et al., 2015). A study of five hospitals in Cyprus (Papastavrou, Andreou, Tsangari, et al., 2014) used the BERNCA survey, which included 20 questions on activities related to care and support, rehabilitation, monitoring and safety. Responses to the survey indicated the extent to which nurses felt able to perform the activities in the past 7 days. Responses were collected on a four‐point Likert‐type scale, and a “rationing score” was derived from the average sum of all items. A high degree of rationing was negatively associated with all five dimensions of patient satisfaction (Papastavrou, Andreou, Tsangari, et al., 2014). Schubert et al. applied the same BERNCA survey within 118 acute hospital units in Switzerland and demonstrated a 37% reduction in the odds of patients reporting satisfaction with the care they received (*p* = .08) with each 0.5 increase in the rationing score (Schubert et al., 2008). A smaller study (Ausserhofer et al., 2013) of 35 Swiss hospitals used the BERNCA‐R survey (which extends the original BERNCA instrument from 20–32 items and adds the statement *Not required* to the responses options) to capture rationing of care. Nurses reported how frequently they were unable to perform 32 basic nursing activities in the past seven working days due to inadequate time, nurse staffing and/or skill mix. Respondents rated each item on a 5‐point Likert‐type scale (task was not required = 0—often = 4). Results indicated that when patients experienced higher levels of nursing care rationing, they were less likely to recommend the hospital to a family member or a friend (OR = 0.27; 95% CI = 0.11–0.67) (Ausserhofer et al., 2013).

Overall, the evidence shows a consistent detrimental effect of rationing care on patient satisfaction. However, studies used different instruments to capture patient satisfaction, which affects direct comparability of the findings.

### 4.2 Quality of care delivered

Three studies identified from the literature search found a significant association between measures of quality of care and tasks left undone (Table 3). Ball et al. used the RN4CAST survey to examine care left undone in 46 English NHS hospitals. Nurses were asked to report how frequently they were unable to perform any of 13 nursing activities on their last shift due to time constraints. Two measures of “missed care” were derived. The first measure quantified the prevalence of any care being left undone, based on one or more of the activities having been ticked (binary measure). A second score indicated the volume of care left undone, by summing the number of activities ticked per person. The authors showed a significant correlation between the number of items of missed care and nurses perception of quality of care (polyserial correlation = −0.037, *p* < .001) and nurse overall grading of patient safety on their unit/ward (polyserial correlation = −0.40, *p* < .001) (Ausserhofer et al., 2014). Sochalski's study in US acute hospitals used a survey based on a list of seven care activities, and nurses had to indicate which was left undone during their last shift due to lack of time. The results indicated an association between a poor rating of quality of care and the number of tasks left undone (β = −0.20; *p* < .001; Sochalski, 2004). Similar results were reported by Zúñiga et al. in a study of 155 Swiss nursing homes. The authors used BERNCA‐NH (Adapted for Nursing Homes) 19‐item scale. Care workers were asked how often in the last 7 days they could not conduct necessary care activities due to lack of time or high workload. Items were rated on a 5‐point Likert‐type scale, and the mean score per subscale was calculated. The study found that nurses reported a better quality of care when the amount of implicit rationing of care, rehabilitation and monitoring (i.e., a subscale of the BERNCA instrument) was lower (OR 0.34; 95% CI 0.24–0.49), and when less instances of rationing social care were perceived to have occurred (OR 0.80; 95% CI 0.69–0.92) (Zúñiga et al., 2015).

### 4.3 Clinical outcomes

Six studies reported associations between missed care, and one or more clinical outcomes, mainly medication errors; bloodstream infections; pneumonia; UTIs; nosocomial infections; patient falls; pressure ulcers; critical incidents and quality of care; and patient safety. Five of the studies found that missed care was associated with adverse outcomes, but in regard to pressure ulcers, two studies (Ausserhofer et al., 2013; Thompson, 2014) found no significant associations between missed care and the incidence or prevalence of hospital‐acquired pressure ulcers. Results are summarised in Table 4.

**Table 4:** Studies of missed care and clinical outcomes

Study	Context	Associations of missed care and outcomes
Ausserhofer et al. (2013)	Switzerland 132 Units (surgical; medical; mixed surgical–medical units)	Rationing of nursing care was associated with medication administration errors (OR = 2.51; 95% CI = 1.18–5.65); bloodstream infections (OR = 3.01; 95% CI = 1.42–6.34); pneumonia (OR = 2.67; 95% CI = 1.11–6.39)
Carthon et al. (2015)	USA 419 Hospitals (Patients with heart failure. Number of units not specified)	A 10% increase in missed treatments and procedures resulted in patients more likely to experience readmissions within 30 days from hospital discharge (OR = 1.12; 95% CI = 1.06–1.18) The fully adjusted model showed that a 10% increase in missing treatments and procedures was associated with higher odds of patients being readmitted to hospital within 30 days from discharge (OR = 1.07; 95% CI = 1.01–1.13)
Lucero et al. (2010)	USA 168 acute care hospitals (general, vascular and orthopaedic surgical patients. Number of units not specified)	Unmet nursing care needs were associated with wrong medication or dose (*p* < .001); nosocomial infection (*p* < .001); patient falls with injuries (*p* < .001)
Nelson and Flynn (2015)	USA 63 Medicare‐and‐Medicaid certified nursing homes	Administering medications on time (*p* = .000); adequate patients surveillance (*p* = .001); perform necessary treatments and procedures (*p* = .007); comfort/talk with patients (*p* = .008); teach patients and/or families (*p* = .018); document nursing care (*p* = .04); coordinate patient care (*p* = .36) were all associated with the per cent of residents with UTI
Schubert et al. (2008)	Switzerland 118 units (medical, surgical, gynaecology)	Care rationing was a significant predictor of all patient outcomes It was associated with medication error (OR = 1.68; *p* < .005); falls (OR = 2.81; *p* < .001); nosocomial infections (OR = 1.61; *p* < .04); critical incidents (OR = 1.10; *p* < .002); pressure ulcers (OR = 1.15; *p* < .0010)
Schubert et al. (2009)	Switzerland 118 units (medical, surgical, gynaecology)	Three of the identified patient outcomes (nosocomial infections, pressure ulcers, and patient satisfaction) were sensitive to rationing, showing negative consequences at average BERNCA rationing scores of .5 or above (never, rarely or sometimes). Results also showed increases in negative outcomes at rationing average ratings of 1 (rarely)
Thompson (2014)	USA 982 (in 2011) and 1,012 (in 2012) medical, surgical, and medical‐surgical unit	Missed care had no significant direct effects for the pressure ulcer prevalence rates in either 2011 or in 2012

The study by Ausserhofer et al. reported an association between rationing of nursing care and higher nurse‐reported levels of bloodstream infections (OR = 3.01; 95% CI = 1.42–6.34), pneumonia (OR = 2.67; 95% CI = 1.11–6.39) and medication administration errors (OR = 2.51; 95% CI = 1.18–5.65). However, there were no significant effects of rationing care on the incidence of pressure ulcers and urinary tract infections (Ausserhofer et al., 2013). Similarly, a study across 1,291 hospitals in the USA conducted by Thompson found no significant associations of missed care with the prevalence rates of hospital‐acquired pressure ulcers. The author used the National Database of Nurse Quality Indicators survey in 741 US hospitals. This survey reported five activities due on the last shift with a yes/no/not applicable answer. Items were aggregated to the unit level to represent the percentage of nurses on the unit who endorsed each item. Missed care had no significant direct effects for the pressure ulcer prevalence rates either in 2011 or in 2012 (Thompson, 2014).

Results on further clinical outcomes were reported in a study conducted in the USA by Lucero et al. in 168 acute care hospitals. The authors used a survey asking nurses to select from a list of seven care activities that were necessary, but left undone, due to the lack of time during their last shift worked. They concluded that unmet nursing care needs were associated with nosocomial infection (*p* < .001) and patient falls with injuries (*p* < .001) (Lucero, Lake, & Aiken, 2010). The study by Nelson and Flynn in 63 US nursing homes drew on the Multi‐State Nursing Care and Patient Safety Survey, with 12 items asking nurses to indicate which necessary activities were left undone due to the lack of time during their last shift. The authors found a number of missed nursing care tasks associated with a higher likelihood of residents experiencing UTIs. The tasks reported were administering medications on time (*p* = .000); adequate patients surveillance (*p* = .001); performing necessary treatments and procedures (*p* = .007); comforting/talking with patients (*p* = .008); teaching patients and/or families (*p* = .018); documenting nursing care (*p* = .04); coordinating patient care (*p* = .36) (Nelson & Flynn, 2015). A study in eight hospitals in Switzerland found care rationing to be associated with medication errors (OR = 1.68; *p* < .005); falls (OR = 2.81; *p* < .001); nosocomial infections (OR = 1.61; *p* < .04); critical incidents (OR = 1.10; *p* < .002); and pressure ulcers (OR = 1.15; *p* < .0010) (Schubert et al., 2008). A subsequent analysis of the sample from the previous study (1,338 nurses and 779 patients) sought to define a clinically meaningful rationing threshold level and found consistent reports of nosocomial infections, pressure ulcers and patient satisfaction being sensitive to rationing with negative consequences (Schubert, Clarke, Glass, Schaffert‐Witvliet, & De Geest, 2009).

While the evidence originating from nurse reports largely indicates significant associations between missed care and adverse clinical outcomes (e.g., pressure ulcers, medication errors, nosocomial infections), evidence relying on objective clinical data is more mixed, with one study indicating an association between several activities left undone and urinary tract infection. Yet, another study concluded that there was no association between missed care and pressure ulcers. However, these studies derived from diverse contexts, and missed care was captured with different surveys, and as seen in Table 1, their validity was assessed as moderate or weak.

### 4.4 Missed care, readmissions and mortality

Overall four studies explored the association between missed care, readmissions and mortality. They are summarised in Table 5. A large study of 419 hospitals in the USA by Carthon et al. relying on the Multi‐State Nursing Care and Patient Safety Survey showed that a 10% increase in missed treatments and procedures was associated with patients more likely to experience readmissions within 30 days of hospital discharge (OR = 1.12; 95% CI = 1.06–1.18). When the analysis was adjusted for the quality of the work environment, the effect of missing essential nursing was no longer a significant predictor of readmission, except for missing treatments and procedures, which still showed high odds for patients being readmitted to hospital within 30 days of discharge (OR = 1.07; 95% CI = 1.01–1.13) (Carthon, Lasater, Sloane, & Kutney‐Lee, 2015).

**Table 5:** Study of missed nursing care, readmissions and mortality

Study	Context	Associations of missed care and outcomes
Ambrosi et al. (2016)	Italy 12 medical units	There was no association between missed nursing care and inpatient mortality (RR = 0.98; 95% CI = 0.93–1.04)
Carthon et al. (2015)	USA	A 10% increase in missed treatments and procedures resulted in patients more likely to experience readmissions within 30 days from hospital discharge (OR = 1.12; 95% CI = 1.06–1.18) The fully adjusted model showed that a 10% increase in missing treatments and procedures was associated with higher odds of patients being readmitted to hospital within 30 days from discharge (OR = 1.07; 95% CI = 1.01–1.13)
Lucero et al. (2010)	USA 168 acute care hospitals (general, vascular and orthopaedic surgical patients. Number of units not specified)	No association was found between unmet nursing care needs and 30‐day mortality (OR = 0.99; 95% CI = 0.89–1.10)
Schubert et al. (2012)	Switzerland Medical, surgical or gynaecological units (numbers not specified	Patients treated in the hospital with the highest rationing level were 51% more likely to die than those in peer institutions (adjusted OR: 1.51; 95% CI: 1.34–1.70)

Three studies reported associations between missed care and patient mortality. In their study comparing two groups of acute hospitals in Switzerland (*n* = 8 sample; *n* = 71 comparator), Schubert et al. reported that patients admitted to hospitals with the highest level of care rationing (i.e., BERNCA score 1.11–1.40) had a 51% increase in the odds of death compared to those patients hospitalised in the comparison group consisting of 71 out of 352 acute hospitals and specialised clinics in Switzerland (i.e., BERNCA score 0.51–0.80) (OR = 1.51; 95% CI = 1.34–1.70) (Schubert et al., 2012). However, overall levels of inpatient mortality (2.7% vs. 2.8%) and emergency admissions (45.7% vs. 47.4%) were similar for both groups of hospitals. Ambrosi et al. conducted a secondary analysis of data collected in 12 Italian hospitals with the aim of identifying factors associated with in‐hospital mortality of patients >65 years old. They used the MISSCARE survey, where nurses and nurse aides reported the frequency of missing 24 nursing interventions during their last shift on a 5‐point Likert‐type scale (1 = never—5 = always). The analysis showed a statistically significant difference between the groups of patients who died or survived (average missed care score = 51.5% in deceased patients and 52.6% in surviving patients, *p* = .04); however, when stepwise logistic regression analysis was performed, no associations were observed between missed nursing care and inpatient mortality (Ambrosi et al., 2016). Lucero et al., 2010 after adjusting for patient and ward environment characteristics, found no evidence of an association between unmet nursing care needs and 30‐days patient mortality (OR = 0.99; 0.89–1.10).

Overall, these studies provide insufficient evidence to support an effect of missed care on patient mortality. However, the study that considered a larger and more diverse sample seemed to support the notion of the association between missed care and in‐hospital mortality.

## 5 DISCUSSION

In summary, the evidence we reviewed indicates an association between missed care and patient outcomes, albeit tenuous in some instances. A number of studies provide evidence in two major categories of patient outcomes negatively affected by omissions of care: patient satisfaction and clinical outcomes. Patient satisfaction was negatively associated with missed care in four studies. Clinical outcomes affected by missed care, as reported in nine studies, included pressure ulcers, medication errors, nosocomial infections, patient falls, critical incidents, 30‐day hospital readmission and mortality. Although most studies controlled for patient case mix, and hospital and nurse characteristics, differences in the context in which the studies took place (e.g., hospital vs. nursing home) or units included in the studies (e.g., medical, surgical and gynaecology) create potential limitations to the generalisability of the findings.

As with hospital studies, research conducted in nursing homes reports that omission of nursing care activities affects the probability of residents experiencing UTIs and the nurses’ ability to perform certain tasks (i.e., administer medication on time, adequately monitor patients, or perform necessary treatments and procedures).

Despite it being essential to patient safety, surveillance has been reported along with other nursing activities (i.e., ambulation, oral hygiene) that are frequently missed in hospital settings (Osborne et al., 2015). While we found mixed evidence about the relationship between nurse‐reported measures of missed care and mortality, the potential of such negative outcome calls for an in‐depth look of the issues surrounding missed care in the form of inadequate patient surveillance and its consequences. Early identification of physiological deterioration has been recognised as one of the factors associated with preventable hospital deaths (Luettel et al., 2007; Smith, 2010) which relies on timely and adequate patient monitoring. Technological solutions in the form of patient surveillance systems that enable healthcare professionals to efficiently monitor patients and identify those who require the most urgent attention may be a solution to surveillance issues. While automated continuous monitoring has not been shown to be associated with reductions in mortality, innovations in intermittent monitoring, including electronic recording with calculation of a risk based early warning score, have been shown to reduce inpatient deaths (Cardona‐Morrell, Prgomet, Turner, Nicholson, & Hillman, 2016; Schmidt et al., 2015). Such automated clinical risk prediction models could support healthcare providers to deploy resources where they are needed most, resulting in improved outcomes and costs (Imison, Castle‐Clarke, Watson, & Edwards, 2016). However, the introduction of a new system that demands time from an already overstretched workforce needs careful planning.

Increasingly, frequency of missed care is being considered as an indicator to assess the quality of nursing care. As reported in one study in our review, the amount of missed care partially mediates the effects of patient‐to‐nurse ratios and work environment on patient recommending the hospital (Bruyneel et al., 2015). However, bias in the instruments available to measure missed care, coupled with the self‐reported nature of most survey data, limits the comparability of findings from studies in the field (Jones, Gemeinhardt, Thompson, & Hamilton, 2016).

Our findings resonate with research that highlights the associations of staffing levels of different nursing staff with patient outcomes and quality of nursing care services (Needleman, Buerhaus, Mattke, Stewart, & Zelevinsky, 2002). This indicates the potential significance of missed care as a consequence of inadequate nurse staffing resources, although the relationship between missed nursing care and mortality is as yet uncertain.

## 6 CONCLUSIONS

This review shows a modest evidence base for a link between missed care and patient outcomes, generated mostly from nurse and patient self‐reported data. To support the assertion that nurse staffing levels and skill mix are associated with adverse outcomes, more research using objective staffing and outcome measures is required. Nursing staff and patients indicate instances where care delivered or received is suboptimal when staffing levels are inadequate. The negative effects on patients in hospital of missing care tasks have, highlight the significance of exploring further the factors that affect the completion of nursing activities. Limiting the occurrence of omissions of care could potentially increase patient satisfaction and decrease the frequency of negative adverse events.

## CONTRIBUTIONS

Study design: AR‐S, CDO, JB, PG; data collection and analysis: AR‐S, CDO, AM, CK, PG; and manuscript preparation AR‐S, CDO, JB, AM, PM, JB, OR, CK, DP, GS, PG.

## APPENDIX 1

### Adaptation of NICE quality appraisal checklist for quantitative studies (National Institute for Health and Care Excellence (NICE), 2014)

**Table:**

Reviewer			
Study full ref			
Design			
Scores	Internal	External	Comments
2	Strong (++)	NA not applicable (rare)	
1	Moderate (+)	NR (not recorded)	
0	Weak (‐)		

Construct	Internal validity	External validity
1. Study design & analysis: cross sectional (−) or allows for cause/effect (exposure precedes outcome time series) (+)/RCT	□	
2. Setting		□
Developed economy and/or comparable health systems ++		
Emerging economy +		
Other −		
2.1 Is the eligible population/area representative of the source population or area?		□
Single hospital (−)		
Consider whether hospitals potentially included in the study are representative of acute general hospital emergency departments nationally or a large sub‐national unit (e.g., US state) (+1)		
Were the staff/patients eligible to be included in the hospitals representative of all ED admissions (+1) or specific subgroup (−1) or limited time period (−1)		
2.2 Do the selected participants or areas represent the eligible population or area?		□
What % of selected hospitals agreed to participate (+1 for larger studies)		
What % of eligible individuals (staff/patients) participated (60% + is acceptable)? (+1)		
Was the data derived from administrative systems and complete (+1) or		
Were the inclusion or exclusion criteria explicit and appropriate?		
3. Were the main measures and procedures reliable?	□	
Were main measures subjective (−1) or objective (++ for completely objective measures)		
How reliable were measures (e.g., inter‐ or intra‐rater reliability scores)? +1 for evidence of reliability		
Where relevant. was there any indication that measures had been validated (e.g., validated against a gold standard measure or assessed for content)		
3.1 Were the measurements complete?	□	
Were all or most of the study participants who met the defined study outcome definitions likely to have been identified? (++ for mortality, + for other PSIs collected using clearly defined methods, − if abstracted from discharge abstracts)		
4. Was the study sufficiently powered to detect an effect (if one exists)?		□
Were there sufficient units/hospitals/wards/patients to give variation and enough patients to detect effects		
Large multi‐hospital (20+) studies (state/national/international) with administrative data ++		
Smaller studies/single hospital with large numbers of patients (000,000) +		
Other—look at confidence intervals/sample size give ( −) if unclear that results are sufficiently precise		
5. How well were likely confounding factors identified and controlled?		
For main patient/staff outcomes, was there patient/staff level risk adjustment e.g., for AGE, (patient) DIAGNOSIS and COMORBIDITY(+ or ++) as appropriate. IT'S/RCT consider +1		
5.1 Were the analytical methods appropriate?	□	
Was there adjustment for clustering of data within hospitals? (+ 1), Where relevant was there control for ward/hospital characteristics (+1)		
5.2 Was the precision of association given or calculable? Is association meaningful?	□	
Were confidence intervals or *p* values for effect estimates given or possible to calculate?		
Were CIs wide or were they sufficiently precise to aid decision‐making? If precision is lacking, is this because the study is under‐powered? If correlations between observations and workload how precise is the prediction?		
Overall score	□	
5.3 Are the study results internally valid (i.e., unbiased)?		
How well did the study minimise sources of bias (i.e., adjusting for potential confounders)?		
Were there significant flaws in the study design?		
5.4 Are the findings generalizable to the source population (i.e., externally valid)?	□	
Are there sufficient details given about the study to determine if the findings are generalizable to the source population?		
Consider: participants, interventions and comparisons, outcomes, resource and policy implications		
